# Feature Papers in Ecotoxicology

**DOI:** 10.3390/jox15030062

**Published:** 2025-04-26

**Authors:** Valerio Matozzo, Maria Gabriella Marin

**Affiliations:** Department of Biology, University of Padova, Via Bassi 58/B, 35131 Padova, Italy; mgmar@mail.bio.unipd.it

Ecotoxicology has progressively evolved as a key scientific discipline for evaluating the impact of both traditional and emerging contaminants on ecosystems. Initially focused on the toxic effects of conventional pollutants such as heavy metals and pesticides, this field has expanded to address a wide array of emerging contaminants, including pharmaceuticals, personal care products, and microplastics, which pose complex and often subtle ecological risks [1,2]. Advances in molecular and omics technologies have enhanced the sensitivity and specificity of ecotoxicological assessments, allowing for the detection of sub-lethal and long-term effects on non-target species and communities [3]. As regulatory frameworks increasingly incorporate ecotoxicological data, this discipline plays a vital role in environmental risk assessment and the development of sustainable pollution management strategies [4].

The collection of research and review papers presented in this Special Issue, “Feature Papers in Ecotoxicology”, highlight recent developments in assessing the ecological and biological impacts of various contaminants, including pesticides, nanoplastics, rare-earth elements (REEs), bisphenols, per- and polyfluoroalkyl substances (PFASs), nanomaterials, and endocrine-disrupting chemicals, in various species. These studies offer critical insights into the ecotoxicological mechanisms underlying pollutant exposure and help in identifying key knowledge gaps that must be addressed to improve environmental protection strategies.

A significant theme emerging from this Special Issue incorporates the unintended ecotoxicological consequences of materials designed to provide safer alternatives to traditional pollutants. While biopesticides are generally perceived as environmentally friendly, research on *Allium cepa* has shown that even natural and microbial formulations can exert cyto-genotoxic effects comparable to conventional pesticides, raising concerns in relation to their large-scale application. This underscores the necessity for thorough pre-market testing and long-term ecological impact studies.

Nanoplastics, another growing threat, have been found to affect marine species. The presence of additional environmental stressors, such as salinity fluctuations, can exacerbate the toxicity of contaminants such as REEs, affecting reproductive success and population dynamics in marine species. Similarly, the combination of bisphenol A (BPA) with polystyrene nanoparticles suggests that multiple stressors can have compounding effects, potentially amplifying their toxicity beyond individual exposure outcomes.

The persistence of PFASs in the environment continues to raise concerns due to their bioaccumulative properties and adverse effects on cellular and neuronal function. These findings reinforce the urgency of investigating long-term exposure impacts and developing viable remediation strategies. Likewise, bioaccumulation pathways through food webs, as observed in bivalves consuming bisphenol-exposed microalgae, highlight the need to assess risks associated with the trophic transfer of chemical pollutants.

Innovative experimental models, such as three-dimensional (3D) fish hepatocyte cultures, are proving to represent valuable tools for replicating in vivo responses to contaminants. These cell models provide new opportunities to study the molecular and biochemical effects of pollutants, facilitating more ethical and efficient toxicity screening. Similarly, studies on engineered nanomaterials, such as ZnS quantum dots, reveal how nanoparticles can disrupt primary producers such as microalgae, potentially altering entire aquatic ecosystems.

While these studies advance our understanding of ecotoxicological threats, they also expose critical knowledge gaps. There is a pressing need for the following work:

- Long-term and multigenerational studies to assess chronic and transgenerational effects of pollutants;
- Integrated multi-stressor assessments to understand how environmental factors interact with contaminants;
- Improved regulatory frameworks that account for emerging pollutants and their complex environmental interactions;
- Scalable and sustainable remediation techniques, for example, assessing the potential of bamboo-derived biochar for pollutant removal, which remains underexplored.

As environmental pollution continues to develop, so too must our approaches to mitigating its effects. The research presented in this Special Issue highlights both the urgency and the possibility of advancing ecotoxicology through interdisciplinary collaboration, innovative methodologies, and forward-thinking policies. Addressing these challenges will not only enhance scientific knowledge but also drive the implementation of meaningful environmental protection measures in an increasingly complex and contaminated world.

## Data Availability

Not applicable.
